# A Systems Biology Approach to Investigating Apoptotic Stimuli as Effectors of Cell Metabolism: Practical Application of Top-Down Control Analysis to Attached Neurons

**DOI:** 10.3390/ijms10020702

**Published:** 2009-02-23

**Authors:** Mika B. Jekabsons

**Affiliations:** Department of Biology, University of Mississippi / 110 Shoemaker Hall, University, MS 38677 USA; E-Mail: jekabson@olemiss.edu; Tel. +1-662-915-3998; Fax: +1-662-915-5144

**Keywords:** Apoptosis, glycolysis, mitochondria

## Abstract

Reduced glycolytic and mitochondrial respiration rates are common features of apoptosis that may reflect key events contributing to cell death. However, it is unclear to what extent the rate changes can be explained by direct alterations in the kinetics of the participating reactions, as changes in the concentrations of intermediates also affect reaction rates. Direct kinetic changes can be identified, ranked, and compared to the indirect effects mediated by the intermediates using top-down control analysis. Flux changes that are explained primarily by direct effects are likely to be prime targets of the pathways that signal death, and thus important contributors to apoptosis. Control analysis concepts relevant to identifying such effects are reviewed. Metabolic flux measurements are essential for this approach, but can be technically difficult, particularly when using adherent cells such as neurons. A simple method is described that renders such measurements feasible.

## 1. Introduction

For newly differentiated neurons to survive during development, the mitochondrial apoptotic pathway, a default death program characterized by nuclear condensation, DNA fragmentation, and membrane blebbing, must be suppressed [1,2]. This suppression is primarily dictated by trophic factors external to the neuron- excitatory neurotransmitters, such as glutamate, and growth factors such as insulin-like growth factor-1 and brain-derived neurotrophic factor [3–5]. Trophic factors act to affect the levels/activities of pro- and anti-apoptotic proteins such as Bax, Apaf-1, and IAPs [6,7], and possibly, as discussed in this article, the activities of enzymes involved in the uptake and processing of glucose. Neurons that survive to become a functional part of the mature nervous system can die inappropriately due to a variety of factors that may overcome this suppression. Inappropriate apoptosis likely contributes to pathologies such as neurodegenerative diseases [3,8,9] and delayed neuronal death in the hours following stroke [10,11]. In some cases, conditions that promote neurodegeneration act by inhibiting one or more reactions involved in energy metabolism (i.e., those responsible for producing ATP). Oxidative stress, a condition commonly associated with apoptosis and neurodegeneration, negatively impacts mitochondrial function. Diminished glutathione levels, a manifestation of oxidative stress, inhibits mitochondrial ATP export [12] and complex I activity [13]. Complex I defects may be particularly important to development of Parkinson’s disease [13]. Mitochondrial accumulation of α-synuclein, implicated in familial Parkinson’s, may also impair complex I [14]. Complex I restriction by chronic, low-dose rotenone administration induces Parkinson’s-like symptoms in rats [15]. Aggregated β amyloid protein and tau, the hallmarks of Alzheimer’s disease, may infiltrate membranes to potentially affect glucose transport and mitochondrial function [16–20]. In Huntington’s disease, mutant huntingtin protein may also impair mitochondrial function [21–23]. These effects may promote apoptosis by facilitating mitochondrial cytochrome c release. While such instances of direct metabolic inhibition have been identified in pathologies associated with inappropriate apoptosis, it is unclear if physiological apoptotic triggers act in the same way.

Reduced glycolytic and mitochondrial fluxes are common features of physiological apoptosis induced by limited trophic factor support, yet the basis for the change in flux is uncertain. Growth factor restriction is associated with reduced glycolytic flux in non-neuronal cells, possibly due to inhibition of one or more glycolytic steps [24–28]. Glutamate stimulates cerebellar granule neuron respiration [29–31], implying that limited availability reduces metabolism. While these observations are consistent with metabolism contributing to physiological apoptosis, the underlying processes that bring about a flux change have not been comprehensively considered. Growth factor deprivation may alter the kinetics of a particular reaction, but the consequences of this on the rate at which a pathway operates (i.e., its flux) is often difficult to ascertain. Stated another way, where a change in flux has been observed, the extent to which it can be attributed to a particular reaction is often unclear. Metabolic control analysis has shown that the flux through most pathways is controlled by a number of steps such that a change in activity of one reaction often has small effects on the overall rate [32]. Metabolic flux varies in most cells primarily because of variations in ATP demand which, as described later, indirectly affect reaction rates through changes in the concentrations of the intermediates in the pathway. A flux change brought about in this way is arguably unimportant for signaling apoptosis, yet such changes are usually not considered. The goal of this article is to review the utility of top-down control analysis [33] as an approach to identifying and ranking both direct kinetic and indirect intermediate changes responsible for perturbing a steady-state. This approach considers blocks of enzymes as a single reaction, thereby simplifying cellular complexity to an experimentally workable system. The analysis quantifies how the blocks respond to a select group of intermediates, and the control the blocks have in establishing a steady-state. This information can then be used to assess the different routes through which an apoptotic stimulus brings about a change in steady-state flux. Cerebellar granule neurons are introduced as a model system in which to study apoptosis. This is followed by a review of concepts important to control analysis, and a description of how the concepts can be practically applied to neurons.

## 2. Metabolic changes during cerebellar granule neuron apoptosis

Cerebellar granule neurons (CGNs) isolated from newborn rats mature *in vitro* when cultured with fetal bovine serum as a source of growth factors, and non-physiologically high extracellular K^+^ (typically 25 mM) to mimic tonic excitatory synaptic input [34–37]. When deprived of these trophic factors, CGNs display the classic morphological and biochemical signatures of death by apoptosis-nuclear condensation [34,38], DNA laddering [34,39], phosphatidylserine exposure [38], Bax activation [6,40], and cytochrome c redistribution [39,41]. High K^+^ depolarizes the plasma membrane potential (ΔΨ_p_ ≈ −35 mV) [42] sufficiently to open voltage-gated L-type Ca^2+^ channels. This approximately doubles the cytoplasmic Ca^2+^ concentration ([Ca^2+^]_c_) from the low K^+^ (i.e., 3–5 mM) state, where the majority of L-type Ca^2+^ channels are closed (ΔΨ_p_ ≈−75 mV) [43–45]. Physiologically, glutamate is the prime determinant of plasma membrane Ca^2+^ permeability, but is impractical to use *in vitro* because it is metabolized and can additionally induce death with extended exposure at moderate concentrations. Thus, *in vitro* neurons exposed to low K^+^ can be viewed as similar to those *in vivo* that fail to establish active excitatory synaptic contacts.

The change in [Ca^2+^]_c_ from high to low K^+^ significantly affects CGN metabolism [38]. Specifically, mitochondrial oxidative phosphorylation rate falls 40%, and glycolytic ATP synthesis rate decreases approximately 20% [38]. The changes are dependent on the [Ca^2+^]_c_, as the effect can be reproduced by omitting Ca^2+^ from high K^+^ buffer [38], a condition that lowers [Ca^2+^]_c_ to a level similar to that in low K^+^. Lower [Ca^2+^]_c_ could affect metabolism in two ways. First, the kinetics of the enzymes may be affected, either by Ca^2+^ directly (e.g., activation of tricarboxylic acid cycle dehydrogenases and thus pyruvate oxidation with Ca^2+^ uptake into the matrix), or by the phosphorylation state of the enzymes or regulatory proteins that affect them, because of altered Ca^2+^-dependent kinase (e.g. calcium-calmodulin-dependent and mitogen activated protein kinases) activity. Second, ATP demand may decline as a result of decreased plasma membrane Ca^2+^ cycling which, as described below, will alter enzyme activity through changes in the concentrations of intermediates. It is unclear to what extent each might contribute to the changes in glycolytic and oxidative phosphorylation fluxes.

Superimposed on the Ca^2+^-dependent changes are those mediated by growth factor deprivation, which acts synergistically with low K^+^ to facilitate CGN apoptosis [41,46,47]. Physiologically, this may occur as the expanded neuron population competes for a limited pool of growth factors [3,4,48–50]. Activation of receptor tyrosine kinases (RTKs) by growth factors has been shown to stimulate TRP channels [51], thereby potentially contributing to the Ca^2+^-dependent mechanisms. More importantly, RTKs stimulate a number of survival kinase pathways, the most important of which in many cells is phosphatidylinositol-3-kinase (PI-3K) and its downstream target protein kinase B/Akt. The PI-3K/Akt pathway may target steps in glycolysis, potentially explaining the flux changes observed during apoptosis [24,52]. Interleukin withdrawal induces apoptosis and suppresses glycolytic flux in hematopoietic cells [26,27]. Insulin-like growth factor-1, which can rescue CGNs and other primary neuronal cultures from apoptosis through PI-3K/Akt [47], stimulates glycolytic flux in a neuronal cell line [28]. Inhibition of hexokinase/glucokinase [28,52–55], phosphofructokinase [26] and glucose transport [27,52,56] has been suggested to explain the suppression of glycolysis observed upon growth factor withdrawal. The phosphorylation state of the pro-apoptotic protein BAD may be one of the links between apoptosis and metabolism, as it has been shown to interact with hexokinase [55] and phosphofructokinase [26] in a growth factor-dependent manner.

The emergence of glycolytic suppression as a potential contributor to apoptosis complements studies showing abnormally high flux rates in cancer cells, many of which are resistant to apoptosis. However, it is crucial to know the extent to which the decreased flux observed during apoptosis can be attributed to changes in (1) the kinetics of the glycolytic reactions and (2) the concentrations of intermediates to which the glycolytic enzymes respond. If the flux decreases primarily because of altered reaction kinetics, then this could have important consequences on mitochondrial function and ATP levels, whereas if it decreases due to changes in the concentrations of key intermediates, then the apoptotic signaling must be acting elsewhere in the system. In this respect, it is important to view glycolysis as a reaction embedded within a larger pathway (whose principle function is to produce ATP) so that potential signaling routes that do not target glycolysis but nonetheless affect its flux can be considered. By broadening the scope of metabolism to include those reactions connected to glycolysis through key intermediates, a more comprehensive picture of the actions of apoptosis will be achieved.

Glycolytic flux is strongly affected by ATP levels, which simultaneously affect the rates of multiple reactions. The balance of rates of ATP utilization and production establishes the ATP level. Most studies have not determined the extent to which ATP utilization changes with apoptosis, and, if so, how much the change can explain the suppression of glycolysis through effects on ATP levels. Granule neurons are a good example, where decreased Ca^2+^ cycling and subsequent decreased ATP utilization may explain much of the decrease in glycolysis. Increased ATP could also occur by activation of the mitochondrial phosphorylation reactions. Thus, the processes altering glycolytic flux could be through direct actions on the glycolytic enzymes, or signaling that acts elsewhere to influence key metabolites, or a combination of these.

Assume that at least some of the decrease in glycolysis can be explained by direct inhibition of one or more glycolytic reactions. It would be useful to know how much this affects mitochondrial substrate oxidation and ATP synthesis. Glycolytic impairment has been implicated in restricting cellular ATP supply, both through reduced substrate level phosphorylation and by restricting mitochondrial substrate supply, thereby promoting mitochondrial dysfunction. However, in most cells glycolysis accounts for considerably less than half of total ATP synthesis, so decreased glycolysis may have little effect on ATP levels. It has been suggested that mitochondrial ATP synthesis decreases because of substrate limitation, but this has not been rigorously tested. Much of the glucose that enters glycolysis is not oxidized by mitochondria, but ends up exported from the cell as lactate. It can be argued that impaired glycolysis results in a greater proportion of pyruvate being oxidized by the mitochondria rather than being converted to lactate, resulting in no diminution in mitochondrial substrate supply under physiological conditions. For these reasons, when apoptotic stimuli directly affect glycolysis, it is important to assess the extent to which this functionally alters mitochondrial substrate oxidation and/or phosphorylation. A systems biology approach to metabolism, through the use to top-down control analysis, can help resolve some of these uncertainties.

Direct changes in the kinetics of those reactions which together exert significant control over a pathway flux indicate a concerted action that is likely important to apoptosis. Despite extensive analysis of the early bioenergetic changes occurring in K^+^/serum deprived CGNs, direct kinetic changes and indirect effects mediated through the intermediates were not distinguished [38]. As mentioned above, one primary disturbance elicited by K^+^/serum deprivation is decreased Ca^2+^ cycling and hence decreased ATP utilization. This primary disturbance is propagated throughout other reactions within the metabolic network through changes in the concentrations of other key intermediates in the system. Reduced ATP utilization produces a transient imbalance in supply and demand: ATP synthesis rate by glycolysis and the mitochondrial phosphorylating system exceeds utilization, resulting in increased ATP/ADP ratio. Consistent with this scheme, higher ATP levels have been measured after K^+^/serum deprivation [57]. This will inhibit glycolytic and phosphorylating system fluxes through effects on enzymes such as pyruvate kinase and ATP synthase whose activities are affected by ATP levels. As these fluxes decrease, other transient imbalances occur, and the initial disturbance begins to propagate through the system.

Looking one step further in this scheme, reduced glycolytic flux will increase glucose-6-phosphate levels as its rate of production by glucose uptake and phosphorylation reactions exceeds the now lower consumption through glycolysis. The increased concentration of glucose-6-phosphate negatively affects hexokinase and thus the rate of glucose phosphorylation. At the same time, decreased mitochondrial phosphorylation system flux causes transient inequity between production and consumption of the mitochondrial protonmotive force. Proton extrusion by the respiratory chain exceeds influx through the now slightly less active ATP synthase. The protonmotive force increases, resulting in inhibition of the oxidation reactions that produce it.

This series of events illustrates how a direct change in flux through one group of reactions (those that utilize ATP) due to a primary disturbance (such as decrease cytoplasmic Ca^2+^) begins a series of transient inequalities in reaction fluxes that produce and consume intermediates within the energy metabolism pathway. This produces changes in the concentrations of the intermediates connecting them, which then alter the reaction rates of the enzymes. Thus, the initial disturbance propagates throughout the metabolic network until a new steady-state is achieved. Flux changes arising from these indirect changes in the concentrations of key intermediates are probably not important in signaling apoptosis, as these would be changes that occur in the normal course of metabolism of any healthy cell. This description, however, is a qualitative one, where it has been assumed that once a primary disturbance occurs, any flux change elsewhere arises entirely as a result of the changes in the concentrations of intermediates. It is possible that one or more reactions are affected to a greater (or lesser) extent than would be expected simply by the changes in intermediate concentrations. A number of kinases, phosphatases, and/or pro-apoptotic proteins affected by K^+^ /serum deprivation could mediate these effects. Such direct changes would be candidate signaling steps contributing to apoptosis, but they would be obscured because of the indirect changes that may also occur. Regulation analysis is a branch of metabolic control analysis that can be used to distinguish between primary and secondary changes, and additionally provide quantitative information on how strongly different reactions are directly affected [58]. Such an approach, when applied to whole cells using a ‘top-down’ view, would provide a comprehensive description of the sites targeted by apoptotic stimuli.

## 3. Top-down metabolic control analysis

Cells are inherently steady-state systems, meaning that the rate of any given metabolic pathway does not continuously fluctuate with time, but rather tends to remain constant. As an electrical analogy, cells can be viewed as direct current rather than alternating current systems. The steady-state can, and does, vary depending on prevailing physiological conditions (e.g., the presence of a hormone, the workload of the cell, etc.). Control analysis was developed to quantitatively describe the contribution of each reaction to bringing about small variations in the steady-state flux through any given point in a pathway [59–61]. It is based on the ‘connectedness’ of multiple reactions through the shared intermediates. In a simple 2-step pathway, an enzyme E_1_ can exert control over the flux catalyzed by a second enzyme E_2_ through its effects on influencing the concentration of the intermediate to which E_2_ responds. As shown below, the intermediate x produced by E_1_ is the substrate for E_2_.

$$\text{S}\underset{{\text{E}}_{1}}{\to }\text{x}\underset{{\text{E}}_{2}}{\to }\text{P}$$

For simplicity, assume an infinite source of substrate S and infinite sink of product P such that their concentrations do not change with time; since their effects on E_1_ and E_2_ are constant, any change in the reaction rates can be attributed to changes in the concentration of intermediate x. Prior to reaching a steady-state, as E_1_ produces x, its concentration will increase such that (1) E_2_ consumes x at an increasing rate as its concentration approaches the E_2_ Michaelis constant, and (2) E_1_ produces x more slowly because of product inhibition. The rates ultimately converge to establish a steady-state flux, with the kinetics of both enzymes to x determining when this is achieved. Since the kinetics of E_1_ to x partly determines the steady-state concentration of x, which in turn influences the rate of E_2_, then E_1_ will exert control over the reaction catalyzed by E_2_. The flux control coefficient is a measure of this control and is defined as:

(1) $$C_{E_{1}}^{E_{2}}=\frac{dJ_{E2}/J_{E2}}{dv_{E1}/v_{E1}}$$

where *dJ_E_*_2_ is the flux change through E_2_ as a result of an infinitesimal change *dv_E_*_1_ in the rate through E_1_. The E_1_ rate change is denoted *v* to indicate that its rate has been directly manipulated (for example, by changing the amount of active enzyme or by addition of an inhibitor or activator); the E_2_ rate is denoted *J_E_*_2_ to indicate that it is a measured system flux dependent on the concentrations of the intermediates in the system. If E_1_ exerts only partial control over E_2_ flux (that is, *dJ_E_*_2_/*J_E_*_2_ < *dv_E_*_1_*/v_E_*_1_), then E_2_ exerts the remaining control over its own flux, such that $C_{E1}^{E2}+C_{E2}^{E2}=1$. If E_1_ and E_2_ are part of a larger pathway as illustrated below, then the control E_1_ and E_2_ exert over E_2_ flux will also depend on how the remaining enzyme(s) affect the concentrations of pathway intermediates to which E_2_ responds.

$$\text{S}\underset{{\text{E}}_{1}}{\to }\text{x}\underset{{\text{E}}_{2}}{\to }\text{y}\underset{{\text{E}}_{3}}{\to }\text{P}$$

In this example, the product of the reaction catalyzed by E_2_- intermediate y- is the substrate for a third reaction catalyzed by enzyme E_3_. Assuming y exerts inhibition on E_2_, the kinetics of E_3_ to y will partly determine the concentration of y, and thus the flux through E_2_. Enzyme E_3_ will therefore exert some degree of control over E_2_ through its effects on establishing the concentration of y, and in so doing lessens some of the control by E_1_. The concentration of x is no longer the only influence on E_2_; the product y is now explicitly considered as also affecting E_2_. As the pool of intermediates that influence a reaction flux increases, so too will the number of reactions involved in controlling the flux because of their effects on determining the concentrations of the intermediates. If, in one particular steady-state, a reaction has more influence on dictating the concentrations of intermediates than in another steady-state, then it will exert more control in the former state, while the remaining enzymes collectively exert less control. Flux control coefficients are therefore properties of the system operating at a given steady-state and are unique to that state.

From this example, it is apparent that multiple reactions in a pathway have the potential to control the flux through a reaction of interest in the same pathway because of their effects in determining the concentrations of the intermediates. Quantifying the kinetics of the reactions to the intermediates is central to control analysis, as the balance of these responses determines the control pattern that emerges in a steady-state. In control analysis, the responsiveness of an enzyme to an intermediate is given by the elasticity coefficient:

(2) $$\varepsilon_{x}^{E2}=\frac{dv_{E2}/v_{E2}}{dx/x}$$

where *dv_E_*_2_ is the rate change through enzyme E_2_ as a result of an infinitesimal change *dx* in the concentration of intermediate x. Here the rate of the reaction catalyzed by E_2_ is designated *v* rather than *J* to emphasize that elasticities denote how the reaction rate of an isolated enzyme is affected by a small concentration change in one intermediate while all other intermediates are held constant. For elasticities to be applicable to the conditions existing within cells, each must be determined in the presence of the physiological concentrations of all other relevant intermediates and effectors. As with control coefficients, the elasticities are only valid for the steady-state condition in which they are determined.

Elasticities and flux control coefficients have been determined for limited segments of metabolic pathways but not complete ones due to the enormous experimental effort required. However, Ainscow and Brand [62] have shown that elasticities and control coefficients can be determined for metabolic pathways within cells by reducing the number of explicit steps to be analyzed. This is done by grouping multiple enzymes together as a single step or block, with the blocks linked by a few key intermediates [63]. Known as the ‘top-down’ approach to control analysis, it provides information on the control pattern present within a complex metabolic network operating at a particular steady-state. More importantly, the information obtained can be used to determine how a parameter external to the system (e.g., the presence of an apoptotic stimulus) brings about a change in system flux through its direct effects on the different blocks in the system [64,65]. The direct effects are quantified as “integrated elasticities” (see below), which can be used with the flux control coefficients to identify the extent to which a flux change through one block (such as glycolysis) occurs indirectly because of the parameter acting directly on the other blocks in the pathway [64]. Since this approach both identifies sites within a complex pathway that are directly affected by a parameter, and additionally provides information on how strongly each affected site contributes to the flux change through the other reactions in the system, it has the potential to provide a comprehensive understanding of how apoptosis acts to bring about energy metabolism changes. The information will be useful in directing research efforts toward the enzymes within a particular block which may be targeted by apoptosis signaling.

**Figure 1:**
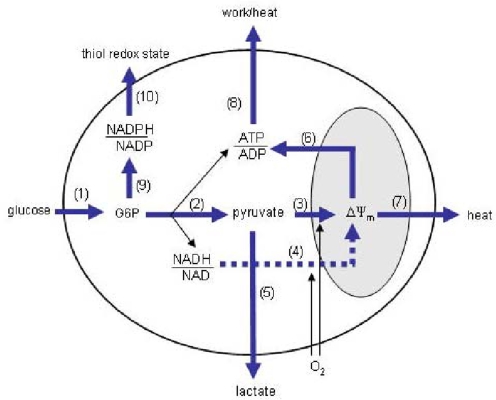
A simplified model of energy metabolism. Reactions involved in producing and consuming ATP have been grouped into 10 blocks separated by 6 intermediates. The blocks are numbered as (1) glucose transport and phosphorylation (GTP), (2) glycolysis (GLY), (3) pyruvate oxidation (PYR), (4), NADH oxidation (NAO), (5) lactate production (LAC), (6) mitochondrial phosphorylating system (PHO), (7) mitochondrial H^+^ leak (PRL), (8) ATP consumers (ATC), (9) pentose phosphate pathway (PPP), and (10) NADPH oxidation (NPO). G6P: glucose-6-phosphate; ΔΨ_m_: mitochondrial membrane potential.

Energy metabolism can broadly be defined as those reactions involved in the production and consumption of ATP. Evidence to date suggests that the glycolytic and/or mitochondrial reactions are targeted by apoptosis signaling, so a relatively detailed division of energy metabolism around these blocks is required. Figure 1 illustrates one way to do this while keeping the system sufficiently simple so that it is experimentally practical.

The blocks are based on those conceived by Ainscow and Brand to investigate the control of hepatocyte energy metabolism [62], with a few modifications. First, unlike hepatocytes, neurons do not store appreciable amounts of glycogen, nor do they release glucose, so the current model contains a glucose transport and phosphorylation (GTP) block rather than glycogen breakdown and glucose release blocks. Second, additional blocks comprising the pentose phosphate pathway (PPP) and NADPH oxidation reactions (NPO) have been included to account for the possibility that glucose diversion through the PPP exerts control over the blocks directly involved in producing ATP. For the analysis to be valid, it is assumed that the blocks communicate only through the explicit intermediates connecting them. That is, the control that one block exerts over flux through a second block only occurs through effects it has on the concentrations of the explicit intermediates. For example, the control PHO may have over GLY flux would occur only through the influence PHO has on establishing the concentrations of the explicit intermediates to which GLY has non-zero elasticities. Products and substrates external to the outer boundary are assumed to have no affect on system fluxes, either because their levels do not change or the changes over the measurement interval do not exert effects on the blocks. The thiol redox state may not meet this criterion, as small variations are likely to influence NPO flux. However, to keep the system relatively simple, the thiol redox state is not explicitly considered; any changes in NPO flux not explained by a change in NADPH/NADP ratio (assuming that this is the only non-zero NPO elasticity) could thus be either a direct effect on the NPO kinetics, or a change in the thiol redox state. Upon a change in the steady-state, the concentrations of the implicit intermediates within the blocks may change, but are assumed to have no influence in establishing the flux through another block. The intermediates through which the blocks communicate are: (1) glucose-6-phosphate (g6p), the product of the GTP block and substrate for glycolysis (GLY) and PPP blocks; (2) pyruvate (pyr), the product of GLY block and substrate for lactate production (LAC) and pyruvate oxidation (PYR) blocks; (3) NADH/NAD ratio, product of GLY block and substrate for LAC and NADH oxidation (NAO) blocks; (4) mitochondrial membrane potential (ΔΨ_m_), product of the PYR and NAO blocks, and substrate for the mitochondrial H^+^ leak (PRL) and phosphorylating (PHO) blocks; (5) ATP/ADP ratio, product of the GLY and PHO blocks, and substrate for the ATP consumer (ATC) block; and (6) NADPH/NADP ratio, product of the PPP block and substrate for the NPO block.

## 4. Determining *in situ* block elasticities to the intermediates

Within cells, reaction block elasticities cannot be directly determined since it is impossible to vary the amount of one intermediate while holding all others constant. They can, however, be calculated using a series of modulations designed to perturb the original steady-state. To see this, first consider a hypothetical isolated reaction block that responds to an explicit intermediate x. The elasticity of the block to x can be estimated by making a finite, small change in x (Δx) and measuring the resulting finite fractional change in velocity.

(3) $$\varepsilon_{x}^{block}\approx \frac{\Delta v_{block}/v_{block}}{\Delta x/x}$$

If the block also responds to a second intermediate y, then the process can be repeated by holding x constant while making a small finite change in y to obtain $\varepsilon_{y}^{blcok}$. If small changes in both x and y are made simultaneously, then the fractional change in velocity is the sum of the changes induced by x and y. By rearrangement of Equation 3, and inclusion of intermediate y, the fractional change is:

(4) $$\frac{\Delta v_{block}}{v_{block}}\approx \varepsilon_{x}^{block}\cdot \frac{\Delta x}{x}+\varepsilon_{y}^{block}\cdot \frac{\Delta y}{y}$$

Now consider the GLY block *in situ*, embedded within the complex energy metabolism pathway and responsive to at least some of the 6 intermediates. If a finite change in the steady-state has occurred due to a change in some physiological parameter that does not directly affect the kinetics of the glycolytic enzymes, then the finite fractional change in flux is approximated by:

(5) $$\frac{\Delta J_{GLY}}{J_{GLY}}\approx \varepsilon_{g6p}^{GLY}\cdot \frac{\Delta g6p}{g6p}+\varepsilon_{pyr}^{GLY}\cdot \frac{\Delta pyr}{pyr}+\varepsilon_{\frac{ATP}{ADP}}^{GLY}.\frac{\Delta ATP/ADP}{ATP/ADP}+\varepsilon_{\frac{NADH}{NAD}}^{GLY}\cdot \frac{\Delta NADH/NAD}{NADH/NAD}+\varepsilon_{\Delta \Psi_{m}}^{GLY}\cdot \frac{\Delta (\Delta \Psi_{m})}{\Delta \Psi_{m}}+\varepsilon_{\frac{NADPH}{NADP}}^{GLY}\cdot \frac{\Delta NADPH/NADP}{NADPH/NADP}$$

If glycolytic flux and the concentrations of all intermediates are experimentally measured, then the elasticities can be solved once five additional equations of the same type are generated. This can be done by manipulating different parameters to perturb the original steady-state of healthy neurons in different ways. This is known as the multiple modulation approach to determining elasticities [66,67]. Each manipulation designed to change the steady-state is referred to as a modulation, and must act at defined sites within the system because flux data from a modulation which directly affects a block (that is, alters its kinetics from the steady-state being analyzed) cannot be used to determine that block’s elasticities. The unknown elasticities are solved by assembling the flux data from modulations that do not directly act on that block. Since finite changes in the intermediates must be measured, the calculated elasticities are an approximation of the true ones, which reflect infinitesimal changes.

## 5. *In situ* targets of apoptotic stimuli: assessing how parameters affect system fluxes

Steady-state systems whose reactions are linked by invariant stoichiometries exhibit matrix relations that allow the flux control coefficients to be calculated from the elasticities, fluxes, and stoichiometries. Theoretical development of the matrix relations, and their experimental application to cells, can be found elsewhere [62,68]. The elasticities and flux control coefficients determined in healthy neurons by the multiple modulation technique are the basis for then understanding how a parameter change, such as the presence of an apoptotic stimulus, acts to perturb the original steady-state. A parameter change acting in unknown ways on the system can alter fluxes by the same means a modulation can- by directly affecting one or more reaction blocks and by indirectly affecting the concentrations of intermediates. Blocks whose kinetics are directly affected will exhibit a flux different from that expected purely from a change in the intermediate concentrations. Such blocks can be identified, and additionally ranked according to the strength that the parameter change (Δq) exerts on the block, by calculating the integrated elasticity (*IE*) coefficient [64] as:

(6) $$IE_{\Delta q}^{block}=\frac{\Delta J_{block}}{J_{block}}-\sum_{i=1}^{n}\varepsilon_{x_{i}}^{block}\cdot \frac{\Delta x_{i}}{x_{i}}$$

where $\varepsilon_{x}^{blcok}$ are the elasticities determined in healthy neurons, and n=number of intermediates. The measured flux change Δ*J_block_/J_block_*, which is known as the integrated response ( $IR_{\Delta q}^{block}$), is potentially the result of both direct and indirect effects, whereas the summation term (an abbreviated form of Equation 5) quantifies only the indirect effects, so the difference represents how strongly a block is directly affected. The parameter change should not cause larger changes in the intermediates than those measured with the modulations that were used to determine the elasticities. If it does, then there will be error in the summation terms, as the true elasticites in the presence of the parameter change will likely differ from the ones determined by the modulation method because at least some of the block responses to the intermediates will be non-linear.

The indirect effects imply that the parameter change exerts direct effects on at least one block in the system to cause the intermediates to change. Thus, at least one block must have a non-zero integrated elasticity to the parameter. The flux change brought about indirectly by each intermediate is referred to as the partial integrated response of a block acting through an intermediate, and is given by the individual summation terms in Equation 6:

(7) IxRΔqblock=εxblock⋅Δxx

In summary, a measured fractional flux change $IR_{\Delta q}^{block}$ is quantitatively partitioned into a direct component $IE_{\Delta q}^{block}$ and an indirect one ∑allxIxRΔqblock, with the indirect component further resolved into effects mediated by each intermediate. Where a block (e.g., block 1) is directly affected by the parameter change such that $IE_{\Delta q}^{block1}\neq 0$, then the effect that has on flux through a second block (block 2) can be determined as the product of the block 1 flux control coefficient over block 2 (determined in healthy neurons) and the integrated elasticity of block 1 to the parameter:

(8) Iblock1RΔqblock2=Cblock1block2⋅IEΔqblock1

This is known as the partial integrated response of a block flux to a parameter change due to the direct effect of the parameter on another block. It gives a different perspective from the partial integrated response acting through an intermediate (Equation 7) by indicating how a direct change in flux through one block affects flux through another because of its actions on changing the concentrations of the intermediates (without specifying which intermediates are important). The effects exerted by glucagon and epinephrine on hepatocytes illustrate the importance of the partial integrated responses in clarifying how a parameter acts to change a flux [65]. Both hormones were found to inhibit glycolytic flux; however, glucagon exerted most of its effect through direct inhibition of glycolysis, whereas epinephrine primarily acted indirectly through its effect on ATP consumption and pyruvate oxidation [65]. These findings emphasize that a measured change in flux must be considered within the context of the metabolic pathway in which it is embedded. The integrated response is the sum of the effects mediated by all blocks directly affected by a parameter change:

(9) IRΔqblock2≈∑i=1rIblockiRΔqblock2

where r is the number of reactions having non-zero integrated elasticities. There may be error in the summation terms because the parameter change may affect the control pattern of the system (i.e., the flux control coefficients can change), which is determined in healthy neurons. There are indications that the control pattern changes are small, as a step-change in Ca^2+^ had very small effects on the control pattern of isolated skeletal muscle mitochondria despite significant flux changes [69]. If gross changes in the control pattern are suspected during application of an apoptotic stimulus, then the control coefficients can be re-determined by the multiple modulation approach, which requires considerably more time and effort. Consequently, if the control pattern in healthy neurons is used, then the partial responses mediated through the different blocks are considered semi-quantitative coefficients. In assessing system responses to a parameter change, the term ‘integrated’ in the coefficients is to indicate that the values are determined for a single step change in a parameter rather than an infinitesimal change, as required for true elasticity and response coefficients.

For this analysis to be valid, the system studied must maintain a steady-state after the parameter change. When the parameter change is a stimulus which induces cell death, is a steady-state possible? Granule neurons deprived of K^+^ and serum, maintain a constant respiration rate for approximately 3–4 hr [38]. This is one indication that CGN energy metabolism maintains a steady-state in the early phases of apoptosis (i.e., prior to >10% of cells exhibiting morphological changes such as nuclear condensation). Another indicator of a steady-state would be the constancy of the intermediate concentrations over the time course of the experiments to be run. Experimental application of these concepts is not trivial, as it requires measurement of the fluxes through each block as well as the concentrations of the intermediates. Standard biochemical assays can be used to determine the intermediate concentrations without much difficulty. However, flux measurements can be particularly challenging when working with adherent cells such as neurons because of the limitations in determining respiration rate, which is crucial for calculating most fluxes. The remainder of this article is devoted to a description of a simple method that can be used to measure the rates of respiration, glucose utilization, and lactate production in the same preparation of neurons.

## 6. Flux measurements in neurons: a practical approach

**Figure 2:**
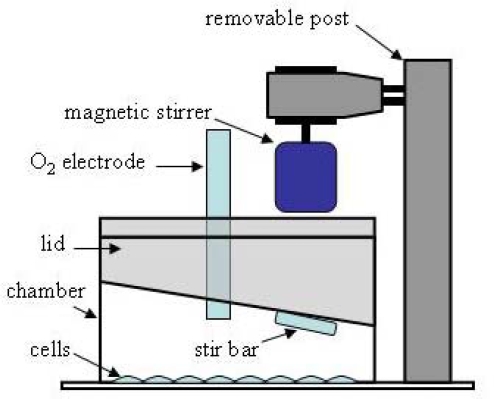
Schematic diagram of a batch-mode approach to measuring the respiration rate of attached cells.

Respiration rate is central to determining fluxes through the blocks which directly produce and consume ΔΨ_m_. Most oxygen electrodes require continual stirring to assure uniform oxygen mixing between the electrode and the buffer into which it is inserted. With adherent cells, this is problematic for two reasons: (1) magnetic stirrers commonly used with oxygen electrodes mix from the chamber bottom where attached cells reside, and (2) as a consequence of (1), most commercially available chambers are designed for cell suspensions. The problem can be circumvented by scraping or trypsinizing cells from their attached substrate, but this is not ideal because of potential damage and loss of attachment-mediated signaling. We recently developed a perfusion method that can be used to measure the respiration of attached cells [29], but it is not suitable for also determining glucose consumption and lactate production so that the fluxes through all reaction blocks can be determined. The ideal setup is one where respiration rate, glucose consumption, and lactate production are measured in the same cell preparation, as this reduces both the number of cells required and the error in calculating the fluxes. A simple setup is described here that fulfills these criteria (Figure 2).

Granule neurons isolated from rat cerebellum are plated in 2-well Lab-Tek chambers (Nunc), with the intent of turning each chamber into a closed system for measuring respiration rate. An acrylic lid was custom fabricated to fit tightly into the top of the chamber. It was made with two ports - one to hold a conventional micro-oxygen electrode (Microelectrodes, Inc.) and one to make additions. Chamber mixing required for proper operation of the oxygen electrode is accomplished with a minineomagnetic stirrer (Instech, Inc.) mounted above the lid to a custom-made removable post that sits in an acrylic base. The neo-magnetic is sufficiently strong to control a micro stir bar through the 1cm thick lid. The lid with oxygen electrode is assembled into the chamber while holding the micro stir bar up against it with the magnet. In this way, the stir bar does not contact the cell layer (Figure 2) and mixes the chamber solution from the top rather than bottom.

**Figure 3:**
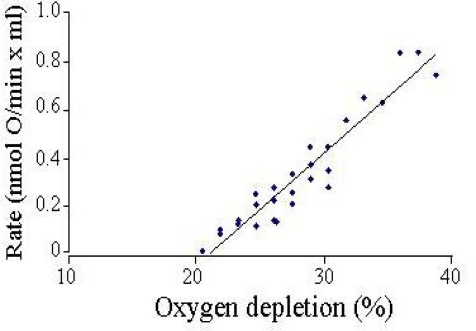
Oxygen back-diffusion rate in 2-well chambers. The cell-free chamber was assembled with buffer (150mM NaCl, 20mM TES pH 7.2, 0.3% albumin) partially depleted of oxygen with nitrogen. The oxygen tension was measured over 2–3 hours. Rates were calculated at 5 nmol O/mL intervals. Data are from 5 independent experiments. The oxygen concentration in air-saturated buffer at 37°C was assumed to be 351 nmol O/mL.

The setup is kept in a temperature-controlled chamber to record the rates at 37°C. Because the chamber walls are made of oxygen permeable plastic, the rate of oxygen back-diffusion was assessed by oxygen-depleting buffer and measuring the increase in oxygen tension over time (Figure 3). To date, few experiments have required corrections for back diffusion, as the cells do not usually deplete the oxygen by more than 15–20% during a typical 20–50 min measurement period. Where corrections have been applied, they are usually less than 5% of the measured respiration rate.

**Figure 4:**
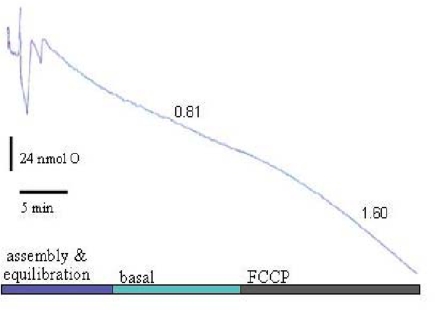
Representative granule neuron oxygen recording using the 2-well chamber system described in Figure 2. Seven million neurons at 37°C were incubated in (mM) 116 NaCl, 25 KCl, 20 TES, 10 glucose, 1.3 CaCl_2_, 1.3 MgCl_2_, 1.2 Na_2_SO_4_, 0.4 KH_2_PO_4_, 0.2 NaHCO_3_, 10% dialyzed fetal bovine serum, pH 7.35. Five to 15 minutes are typically required for a stable baseline to be established once assembled. Basal and 1 μM FCCP respiration rates are expressed in nmol O/min x 10^6^ cells.

Back-diffusion rates were detectable only when >20% oxygen was depleted. In this configuration, neurons in high K^+^/serum buffer exhibit respiration rates similar to that measured by the perfusion method [29,38], and respond predictably to protonophore (e.g., FCCP) addition, a modulation which directly increases flux through the proton leak block (Figure 4).

**Figure 5:**
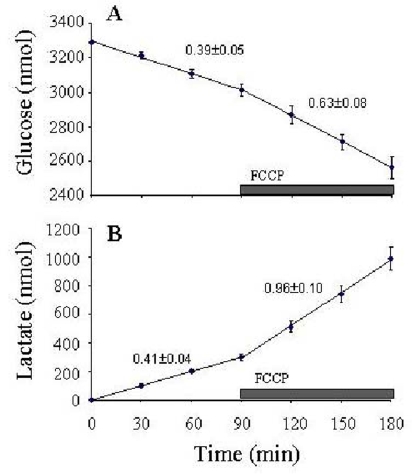
Glucose utilization (**A**) and lactate production (**B**) by CGNs in 2-well chambers. Neurons were incubated with 350 μl high K^+^/serum as detailed in Figure 4. Where indicated, 1 μM FCCP was included to increase H^+^ leak flux. This modulation was propagated through the system via the intermediates to affect glucose uptake and lactate production. Data are the mean ± S.E.M. of three independent experiments. Rates are expressed in nmol/min x 10^6^ cells, as determined from the least-squares regression slopes.

The lid can be removed with the stir bar suspended to it so that the cells remain undisturbed for measurements of glucose utilization and lactate production. The cells are incubated in a minimal volume of buffer over 30–60 min intervals, and the collected samples assayed enzymatically for glucose (using hexokinase/glucose-6-phosphate dehydrogenase/NADP, with NADPH detected spectrophotometrically) and lactate (using lactate dehydrogenase/glutamate-pyruvate transaminase/NAD, with NADH detected fluorimetrically) (Figure 5). Using this approach, it is crucial to run parallel control wells without cells to correct for evaporation, which significantly affects the concentrations with experiments at 37°C. With these three measures performed on the same cells, and knowing the stoichiometries between the blocks, it is possible to calculate the following fluxes: GTP, GLY, LAC, PYR, NAO, PPP, and NPO (Table 1). Additional respiration measurements are required to assess H^+^ leak flux at different ΔΨ_m_ values so that PRL, PHO, and ATC fluxes can be calculated (e.g., see [70]).

## 7. Conclusions

Glycolytic and mitochondrial flux changes have been measured in response to trophic factor withdrawal from a variety of cell types. Measured changes in the activity of one or more glycolytic/mitochondrial proteins have been suggested to account for the flux changes. However, metabolic flux is controlled by a number of interactions, all of which should be considered to obtain a comprehensive picture of the routes through which an effector induces a flux change. These interactions include the responsiveness of the enzymes to the intermediates in the pathway (reflected in the elasticity coefficients), and the influence that each enzyme has on the concentrations of the intermediates, and thus the flux catalyzed by the other enzymes in the pathway (reflected in the flux control coefficients). There are few reports indicating the extent to which a direct kinetic change in a particular protein accounts for a pathway flux change. Moreover, the contribution of indirect effects- changes in the concentrations of the intermediates within the pathways of interest- have not been determined during apoptosis. Identifying kinetic changes brought about directly by an apoptotic stimulus, and the importance of this in causing a flux change, would be useful to understanding the possible role of metabolism in signaling apoptosis. Such a comprehensive picture can be attained using the top-down approach to control analysis. The utility of the approach to understanding how hormones alter cellular metabolism has been described, and its direct applicability to understanding the routes responsible for an observed flux change during apoptosis is apparent. Measurement of respiration rate is central to the determination of metabolic fluxes. This can be difficult when using adherent cells such as neurons, but through the use of a new technique such measurements are feasible.

## Figures and Tables

**Table 1:** Reaction block fluxes in healthy neurons.

Block	Flux	Units
GTP	270 ± 41	pmol glucose/min x 10^6^ cells
GLY	251 ± 13	pmol glucose/min x 10^6^ cells
LAC	406 ± 31	pmol lactate/min x 10^6^ cells
PYR	95 ± 8	pmol pyruvate/min x 10^6^ cells
NAO	95 ± 8	pmol NADH/min x 10^6^ cells
PPP	19 ± 29	pmol glucose/min x 10^6^ cells
NPO	38 ± 57	pmol NADPH/min x 10^6^ cells
PHO	nd	
PRL	nd	
ATC	nd	

Measurement of glucose uptake, lactate production, and respiration rates were used to calculate the fluxes using established stoichiometries between the blocks. Data are mean ± S.E.M. of six independent experiments. PYR and NAO fluxes are identical because a 1:1 stoichiometry exists for pyruvate oxidized and net NADH produced through glycolysis; it is assumed that all of this NADH is oxidized by mitochondria. To calculate PHO, PRL, and ATC fluxes, additional experiments are necessary to determine the kinetics of PRL to ΔΨ_m_ so that the proportion of oxygen utilized for ATP synthesis can be assessed. nd: not determined.
